# Section 12 approval: fit for purpose?

**DOI:** 10.1192/bjb.2019.52

**Published:** 2019-12

**Authors:** David Rigby, Lynsey McAlpine

**Affiliations:** 1 Waltham Forest Older Adult Mental Health Team, North East London NHS Foundation Trust, UK; 2 Medical Education Department, East London NHS Foundation Trust, UK

**Keywords:** Section 12, mental health act, medical education

## Abstract

In light of the increasing numbers of detentions of mentally unwell patients in the UK and the recent review of the Mental Health Act, this editorial seeks to analyse the process of Section 12 approval of doctors from a medical educational perspective. We compare the approval mechanisms with assessments in other specialities and suggest evidence-based improvements. We believe that a rethinking of the Royal College of Psychiatrists' learning objectives for Section 12 approval and the introduction of a summative assessment would improve the knowledge and skills of clinicians performing an important and scrutinised role within our society.

In recent years there has been a dramatic increase in compulsory in-patient psychiatric treatment in the UK. In 2016, National Health Service (NHS) Digital reported a 47% increase in the number of detentions under the Mental Health Act over the preceding decade.^1^ The reasons for this are multifactorial and include reduced access to social care, changes in the provision of psychiatric services and reductions in the number of psychiatric beds. Another factor that must be considered is the Mental Health Act assessment itself. Approval under Section 12 of the Mental Health Act confers the ability to deprive individuals of their liberty and curtail their human rights. This is one of the most life-altering powers a doctor can hold.

There is a danger that clinicians are not equipped with the knowledge and skills to wield this power safely and effectively, which could have a marked effect on the number of detentions under the Mental Health Act. In light of the recent independent review into the Mental Health Act and consequent discussions about what modern mental health legislation should entail, it is imperative that we scrutinise the process for training and approving the clinicians who carry out these assessments. Although the specifics of mental health law vary widely across the world, there are shared fundamental principles and an examination of the training, skills and assessment of professionals carrying out detentions of mentally ill individuals is an important process for psychiatrists to consider.

This article seeks to evaluate these training, approval and revalidation processes for Section 12 doctors and suggests evidence-based improvements.

## The current state of affairs

Under current legislation in England and Wales, the Secretary of State can grant approval under Section 12 of the Mental Health Act, provided the clinician meets the requirements set out in the Act. This responsibility is delegated to local Section 12 panels, which also hold responsibility for the accreditation of induction and refresher courses. A clinician can be approved for a period of up to 5 years; at the end of this period they can apply for revalidation,^2,3^ which is contingent on participation in a section 12 refresher course.

We sent a questionnaire to all 23 providers of Section 12 courses and obtained only 5 responses. Although this is a low response rate, we triangulated this information with other sources to acquire information about key features of the courses. Section 12 induction courses and refresher courses typically last 2 days and 1 day, respectively. The cost of an induction course will range between £200 and £400. Attendance is generally monitored using a sign-in sheet, with no verification of identity. The courses consist of a series of lectures covering the nationally mandated learning objectives (see Box 1). Some courses will include an interactive component such as a case discussion or a quiz. There is no requirement to summatively or formatively assess whether the learning objectives have been met.

Box 1 Section 12 course learning objectives^3^

- To have a broad understanding of the provisions contained in Part 2 of the Mental Health Act relevant to the initial detention of a patient under Sections 2, 3 and 4.
- Be able to describe the role of a Section 12(2) doctor and that of others when undertaking a Mental Health Act assessment.
- Understand the meaning of, and be able to refer to, the criteria for detention under Part 2 when making a medical recommendation.
- Be able to describe the guidance contained in the Code of Practice relevant to the role and responsibilities of a Section 12(2) doctor when undertaking a Mental Health Act assessment.
- To use knowledge of the impact that an assessment may have on patients and their carers to inform the approach to an assessment.
- Be able to complete the statutory forms lawfully.

This lack of formative assessment is particularly concerning considering that there is evidence to indicate that there are inadequacies in many psychiatrists' understanding of the relevant legislation. When a random sample of Section 12-approved clinicians in the West Midlands were interviewed, none of them were able to define ‘mental disorder’ as it appears in the Mental Health Act.^4^ In a similar study in Scotland, only 10% of consultant psychiatrists were able to give the statutory definition of mental disorder.^5^ This raises questions about the training, approval and revalidation processes.

On completion of the induction course, prospective Section 12 doctors must undergo an approvals process whereby they must provide two referees, with the requirement that one of the referees is an NHS consultant psychiatrist familiar with the work of prospective Section 12 doctors. In theory, this is an additional check that the prospective clinician has the required knowledge and skills. In practice, there is no framework for how a referee should assess whether the prospective clinician has the complex skills specific to Mental Health Act assessments. Furthermore, it is likely that clinicians seeking Section 12 approval will choose consultants who are also their line managers as referees. Therefore, it would be equally unlikely that the referee would have witnessed the aspiring Section 12 doctor complete a Mental Health Act assessment because of the Mental Health Act's need for an independent doctor.

## Analysis

When analysing the educational effect of these training courses, three questions must be considered: What are the learning objectives? How does the training meet these learning objectives? And how are they assessed?

### What are the learning objectives?

**Fig. 1:**
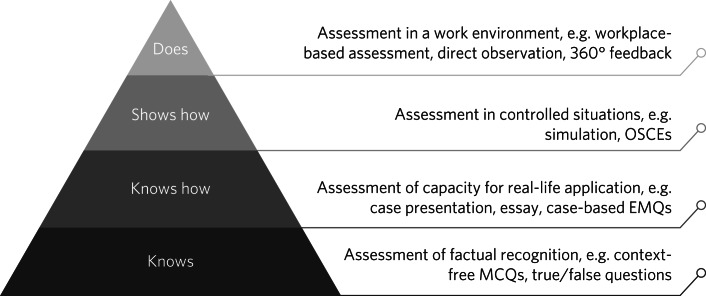
Miller's pyramid^6^ a framework for classification of assessment in medical education, can be used to align assessment with learning outcomes. EMQ, extended matching question; MCQ, multiple choice question; OSCE, objective structured clinical examination.

Miller^6^ described the assessment of clinical skills and competencies as four levels of increasing complexity as shown in Fig. 1. Taking the example of venepuncture: knowing what a blood test is and its indications would satisfy the lowest level of the pyramid. Being able to describe how to take blood would show that the clinician ‘knows how’ to perform the task. Assessing the individual performing a simulated blood test on a manikin would satisfy the third level of the pyramid and observing the competency being performed on a patient would demonstrate proficiency of the highest level on the pyramid.

Biggs and Tang^7^ have aligned the imperative words used to describe learning objectives to levels on Miller's pyramid so that words such as ‘identify’, ‘define’ and ‘describe’ refer to learning objectives at the bottom of the pyramid and ‘perform’ and ‘demonstrate’ are associated with the highest level on the pyramid. By this analysis, the current learning objectives for Section 12 approval courses are only assessing the lowest levels of the pyramid. Indeed, the only practical skills required are ‘completion of the statutory paperwork’ and ‘rectification of errors’. There appears to be a presumption that the clinician will develop the necessary skills elsewhere. This is not necessarily the case; for example, clinicians who have achieved membership of the Royal College of Psychiatrists are eligible to apply for Section 12 approval, but as an international examination, the MRCPsych does not assess UK mental health law.

Performing a Mental Health Act assessment is a complex skill requiring the ability to apply the principles of mental health law to challenging clinical situations; this is not reflected in the current learning objectives, which focus on simple knowledge and skills with relatively little emphasis on higher-order learning.

### How does the training meet these learning objectives?

To motivate prospective Section 12 doctors and support them to achieve these higher-order learning objectives, the learning activities should be closely aligned with these objectives.^8^ At present, Section 12 courses rely heavily on lecture-based teaching, with a focus on conveying large volumes of factual content. This may be problematic because although lectures can be a useful didactic method for imparting factual knowledge, they are less effective at changing attitudes and behaviours. For teaching complex skills, the evidence base supports the use of active learning activities to engage learners in more effective and more sustained learning.^9^

### How is it assessed?

There is an absence of any mandatory assessment in the Section 12 approval process, and this has significant implications. Assessment serves two distinct educational functions: it is used to evaluate whether the learning objectives have been achieved and it is used to drive learning.^10^ The widely held belief that ‘if it's not assessed, it's not important’ is backed up by substantial evidence demonstrating that trainees learn more effectively when they know they will be assessed.^11^ If they are not formally assessed, they will not achieve the learning objectives. The ability to perform a Mental Health Act assessment is a complex skill, requiring a sound knowledge base and extended abstract thinking, and this needs to be reflected in the learning objectives, learning activities and assessment process.

## Our solution

To optimise their educational effect, the design of training courses should be guided by the evidence base. Appropriate learning objectives should be created based on the knowledge and skills required of a Section 12 doctor, and the learning activities should be aligned with these objectives. We propose a teaching model similar to that used in Advanced Life Support (ALS) training run by the UK Resuscitation Council, whereby prospective Section 12 doctors would be given standardised educational material before the course, either as written material or as a series of e-learning modules. This could employ a variety of formats to suit different learning styles, and would allow clinicians the flexibility to work through the material at their own pace. A pre-course self-assessment quiz would allow them to test their level of understanding and identify learning needs, as well as ensuring engagement with the pre-course material.

Instructors should therefore have more confidence that the doctors will have decent levels of working knowledge of the Mental Health Act to build upon during the course. The face-to-face training course would then have scope to focus on higher-order skills such as applying their knowledge of the pre-course material to ‘real-life’ scenarios. Instructors would have flexibility to make their training course unique; for example, by using role play, case discussions and simulated Mental Health Act assessments.

Rather than simply signing an attendance sheet, there should be a formal identification check at the outset of the course. Furthermore, sign-off should be contingent on an end-of-course summative assessment with two key components: a written multiple choice test and a practical assessment such as a case discussion with the instructor. Concerns about failing a substantial number of prospective Section 12 doctors are understandable, but these fears are misplaced: if the assessment is criterion-referenced and aligned with the learning objectives, and the pass mark is determined by an appropriate methodology such as the Angoff^12^ method, the assessment will uphold the minimum standard without failing candidates unnecessarily For context, only 3.4% of candidates fail their ALS training.^13^ With something as important as Section 12 approval, minimum standards must be upheld and summative assessment is the only way to achieve this.

Of course, there are practical and cost considerations when implementing such an assessment. The development of a question bank and determination of an appropriate pass mark would be resource-intensive; nonetheless, it is still feasible and the advantages of incorporating assessment into the training far outweigh the disadvantages. The cost to the delegate of attending a Section 12 approval course are similar to those attending ALS. Therefore it is likely to be financially feasible to implement these changes.

We also propose a modification to the learning objective for Section 12 approval courses to reflect the changes in assessment and the complex nature of the Mental Health Act Assessment as detailed in Box 2.

Box 2 Proposed Section 12 course learning objectives aligned with assessment methods

- **Outline** the provisions contained in Part 2 of the Mental Health Act relevant to the initial detention of a patient under Sections 2, 3 and 4 of the Mental Health Act.
- With reference to the Mental Health Act Code of Practice, **describe** the role of the Section 12 doctor and other participants in a Mental Health Act Assessment.
- **Explain** the criteria for detention under Part 2 of the Mental Health Act.
- **Perform** a holistic assessment of a patient's history and mental state in the context of a Mental Health Act assessment.
- Based on a holistic clinical assessment, demonstrate sound and lawful **application** of the statutory criteria for detention under the Mental Health Act and **justify** the chosen course of action.
- **Demonstrate** lawful and accurate completion of statutory forms.
- **Reflect** on the patient's experience of a Mental Health Act assessment, and how this might affect their mental state and engagement with healthcare services.

## Conclusion

The authority to detain someone against their will is one of the greatest powers that can be granted to our profession, and it is a responsibility that should be taken seriously. Future legislation (and indeed the Royal College of Psychiatrists) should revise the learning objectives of Section 12 courses to mandate a more appropriate standard than the bare minimum set out in the current format of the learning objectives. Efforts should be taken to ensure that this training is evidence-based, developed from sound educational principles and reinforced by appropriate assessment.

## About the authors

**David Rigby** is an ST5 Psychiatrist with Waltham Forest Older Adult Mental Health Team, North East London NHS Foundation Trust, UK. **Lynsey McAlpine** is a Core Psychiatric Trainee and Fellow in Medical Education at East London NHS Foundation Trust and the Centre for Psychiatry, Queen Mary University of London, UK.
